# “I found myself alone” – A phenomenological study of the home care workers' experience during the COVID‐19 pandemic

**DOI:** 10.1111/nhs.12935

**Published:** 2022-03-15

**Authors:** Silvio Simeone, Ercole Vellone, Michele Virgolesi, Madeline R. Sterling, Rosaria Alvaro, Gianluca Pucciarelli

**Affiliations:** ^1^ Department of Clinical and Experimental Medicine University of Catanzaro Magna Graecia Catanzaro Italy; ^2^ Department of Biomedicine and Prevention University of Rome Tor Vergata Rome Italy; ^3^ Emergency and Urgency Department ASL Naples 2 Naples Italy; ^4^ Department of Medicine Weill Cornell Medicine New York New York USA

**Keywords:** care workers, COVID‐19, live experiences, pandemic, phenomenological study

## Abstract

Home care workers (HCWs) are a highly heterogeneous population in Italy in terms of their professional qualifications. HCWs play an important role in helping patients affected by chronic diseases and their families. Although many investigators have studied the lived experiences of family caregivers, few have been conducted “to give a voice” to HCWs and even fewer have examined the experiences of HCWs during the present COVID‐19 pandemic. We investigated the lived experiences of HCWs during the first wave of the pandemic in Italy. Cohen's phenomenological research approach was used to conduct this study. In our study, we enrolled and interviewed 19 HCWs who were female, and most were married, with an average age of 52 years. The participants were enrolled from September 2020 to November 2020, after the first COVID‐19 wave in Italy. Four main themes emerged from the analysis of the data: (1) “I found myself alone”; (2) from invisibility to visibility; (3) a fear of getting sick and infecting others; and (4) “Health or work? That is the question.” Understanding HCWs' lived experiences, especially those related to the COVID‐19 pandemic, is a first step in giving a voice to this important but vulnerable population in the healthcare workforce.

**Key points**

- Numbers of home care workers (HCWs) in Italy are generally migrant women from Eastern European without training and their works were invisible.
- During COVID‐19 pandemic they feel lonely, stressful, and helpless, even though their clients' family members awarded of their importance.
- Feeling of ambivalence and fear are on the way.

## 1 INTRODUCTION

In December 2019, an unknown coronavirus, severe acute respiratory syndrome coronavirus 2, deemed to be the cause of coronavirus disease 2019 (COVID‐19), was identified in China (Li et al., 2020; Wang et al., 2020). The virus quickly spread around the world, causing a global pandemic (The Lancet Infectious Disease, 2020). A specific estimate of the number of victims is difficult as the data change quickly (World Health Organization, 2020). Among the European countries, Italy was one of the hardest hit (Bordi et al., 2020) probably because, compared with the other European countries, Italy has a greater older adult population affected by several chronic diseases (Tur‐Sinai et al., 2020).

Many families required a homecare worker (HCW)'s services also before the COVID‐19 pandemic. Indeed, as described by a report (VITA, 2019), it is estimated that in Italy there are approximately 865,000 HCWs, prevalently female and located in the north and center. Not being a service offered by the state, hiring an HCW has a big impact on families. Indeed, on average a year, the total overall expenditure is estimated at around 7 billion.

Because of the fear of getting infected (de Leo & Trabucchi, 2020), many home‐dwelling older people who needed care hired HCWs, a type of informal caregiver paid for their services by the patient. HCWs provide personal caregiving services, including assisting their clients in activities of daily living, carrying out medically oriented tasks such as helping their older clients take their medications, and in some cases providing companionship to their clients (Grasmo et al., 2021). In Italy, where this study was conducted, HCWs are generally Eastern European women who do not have the qualifications for or formal training to be HCWs.

## 2 BACKGROUND

Studies conducted in the United States have shown that HCWs played a crucial role during the first wave of the COVID‐19 pandemic because of their proximity to clients and their provision of caregiving services (Sterling et al., 2018). Although these people spend a significant amount of time helping their clients manage their chronic diseases, navigating the healthcare system (Ashley et al., 2010; Franzosa et al., 2018) and providing long‐term assistance and post‐hospitalization care for them (Jones et al., 2015, 2017; Madigan, 2008), they are still an invisible and vulnerable workforce and are seldom the focus of research (Sterling et al., 2018).

Indeed, although there have been many studies on the lived experiences of family caregivers (Simeone et al., 2016; Young et al., 2019), most of these studies focused on unpaid caregivers such as family members, friends, and significant others. As for the few studies that have been conducted to give HCWs a voice by analyzing their lived experiences, almost all of them were conducted before the COVID‐19 pandemic (Ayalon et al., 2015; Ohta et al., 2018, 2020; Sterling et al., 2018, 2020). Indeed, to the best of our knowledge, only one study before ours has examined the experiences of HCWs during the present COVID‐19 pandemic (Sterling et al., 2020). This represents a research gap because it is well known that COVID‐19 has had a big impact on people's lives, engendering feelings of isolation and fear in many and affecting their psychological state. The HCWs in Italy may have experienced these more intensely because most of them are migrants and because they have always been considered a high‐risk subpopulation due to their poor health and social conditions and the absence of government regulations to promote their welfare. A full understanding of their lived experiences so far during the present pandemic will help clinicians and researchers develop tailored interventions to protect them and to better understand their relationships with their clients. This lack of attention represents a research gap because it is well known that COVID‐19 has had a big impact on the healthcare workforce.

A full understanding of HCWs' lived experiences during the present COVID‐19 pandemic might help clinicians and researchers develop tailored interventions to protect this population and to help them work better with their clients.

In light of the foregoing, the aim of the study was to analyze the lived experiences of HCWs during the first wave of the pandemic in Italy.

## 3 METHOD

### 3.1 Design

This study used Cohen et al.'s phenomenological research approach (Cohen et al., 2000), which combines descriptive (Husserlian) and interpretative (Gadamerian) phenomenology, because it could enable a deeper understanding of the lived experiences of the participants and of the meanings attributed to such experiences. Cohen's phenomenology combines Heidegger's thought, which can be considered purely descriptive, with that of Gadamer, who contended that hermeneutics is the study of texts. He used that term broadly to mean language. He included not only what people write down but also, more important, what they say and the symbolic activities in which they engage. He stated that to have a world is to have a language: “Our experience of the world is bound to language.” The approach is rooted in a motivation to gain access to the nature of consciousness itself, and the resulting position is based on the meaning of the individual's experience. The same approach has been successfully used by authors in previous studies designed to describe the lived experiences of caregivers and their clients (Simeone et al., 2016, 2018). In addition, phenomenology is particularly useful for examining topics that are complex, ambiguous, and emotionally laden (Smith & Osborn, 2015).

### 3.2 Setting and participants

Convenience snowball sampling was used for participant recruitment. We started recruiting HCWs at employment agencies. Once we contacted the first HCWs, we asked them if they knew any other HCWs. Through this network, we met other HCWs who were asked to be willing to participate in the study. This made it possible to get in touch with a HCW network and facilitate sampling. Participants were enrolled in this study from October to November 2020, after the first wave of COVID‐19 in Italy. The participants' informed consent to participate in this study was obtained before collecting the data.

For inclusion in the study, the participants were required to (1) have been an HCW for at least 6 months before the study; (2) currently be providing care to an older person with or without a chronic disease; (3) understand spoken and written Italian; and (4) consent to participate in the study.

### 3.3 Data collection

Following the chosen research approach, all the researchers involved in this study performed “bracketing” (i.e., putting aside or enclosing in parentheses their preconceptions of the phenomenon that was the object of the study). In the said research approach, it is important for researchers to identify and write down their hypotheses and beliefs about the phenomenon in question (a fundamental technique of “critical reflection”) so that data analysis will not be influenced by the researchers' preconceptions. By carrying out this reflective technique before data collection and analysis, the researchers can deliberately avoid introducing their own biases into the analysis process (Cohen et al., 2000).

After the researcher/interviewer explained the nature and purpose of the study to all participants, the latter chose the date and time of their interviews. In full compliance with the restrictive measures issued to protect Italian citizens' health, this study used video call interviews, which, as described in the literature (Janghorban et al., 2014), can fully substitute for the classic face‐to‐face interviews because they allow for the perceptions of nonverbal language. Video call interviews were conducted using the platform suggested by participants (i.e., Skype, teams or zoom). Each participants had access to a computer, and we did not meet any technical problems with the digital internet connection during the interview.

The interviews were conducted by the first, third, and last authors using a single open‐ended question to guarantee the participants' maximum freedom of expression. In this way, the “world” of each participant became the focus of the research (Polit & Beck, 2018). The participants had previously had no contact with the researcher who conducted the interviews. To ensure that the participants would be able to freely relate and describe their lived experiences, the interviews were held at the venues and times chosen by the participants (where it was assumed they were most comfortable and without the presence of other people), and the interviewer maintained a welcoming attitude during the interview, showing utmost cordiality and impartiality. During the interviews, the researcher wrote field notes (i.e., personal reflections and notes relating to the context and nonverbal language used by the interviewees). Each interview was concluded when the participants expressed that they no longer had anything more to add. Data saturation was considered to have been reached when no more new themes were identified from the participants' interviews (Polit & Beck, 2018). As required by Cohen's methodology (2000), the interview data and the fieldnotes should be converted into digital form as soon as possible after they are gathered. Audiotapes of each interview need to be transcribed verbatim, and the accuracy of the transcription should be thoroughly checked.

This procedure allows you to identify possible themes immediately. The interviews are converted as soon as they are acquired, and this allows to identify the themes early and consequently observe if the themes become redundant or saturated.

All the interviews were only audio recorded and lasted 30–50 min. This decision was made because in a classic phenomenological interview, the methodology suggests to audio‐record only the interviews in order to analyze them and identify any common themes. By video recording the interview, we could no longer guarantee confidentiality and anonymize/de‐identify their data. Information about participants and their clients are reported in Table 1.

**TABLE 1:** Sociodemographic and working characteristics of the homecare workers (*n* = 19)

Code	Gender	Age	Home country	Marital status	Education	Healthcare training	Years in Italy	HCW experience (in years)	Living situation	Uses public transport	Patient gender	Patient age	Patient clinical condition
AZ01	F	54	Russia	Married	High school	No	11	10	House of patient	Yes	M	75	Alzheimer
BV02	F	47	Ukraine	Married	Middle school	No	7	5	House with friends	Yes	M	68	Diabetes, hypertension
CU03	F	49	Moldova	Married	High school	No	9	9	House with friends	Yes	M	67	Dementia
DT04	F	54	Russia	Single	Middle school	No	13	9	House with family	Yes	M	71	Diabetes
ES05	F	56	Bangladesh	Married	Primary school	No	10	7	House with family	Yes	M	63	Stroke, hypertension
FR06	F	59	Italy	Married	Middle school	No	59	15	House with family	Yes	F	73	By‐pass, diabetes
GQ07	F	57	Italy	Married	High school	Yes	57	1	House with family	Yes	M	67	Hypertension, diabetes
HP08	F	51	Moldova	Single	High school	No	10	9	House of patient	No	F	64	Alzheimer, diabetes
IO09	F	52	Ukraine	Married	Middle school	No	7	5	House with friends	Yes	M	77	Dementia, hypertension
LN10	F	48	Moldova	Married	High school	No	9	9	House with friends	Yes	M	71	Chronic obstructive pulmonary disease
MM11	F	54	Moldova	Single	Middle school	No	13	9	House with friends	Yes	F	78	Dementia, diabetes
NN12	F	51	Peru	Married	Middle school	No	8	5	House with friends	Yes	F	75	Parkinson's, Alzheimer
OM13	F	56	Moldova	Married	Middle school	No	12	9	House of patient	No	F	70	Alzheimer
PL14	F	51	Romania	Married	High school	No	11	9	House with family	No	M	68	–
QI15	F	53	Ukraine	Married	Bachelor's degree	No	13	11	House of patient	Yes	M	71	Parkinson's, dementia
RH16	F	46	Ukraine	Married	Middle school	No	7	5	House with friends	No	M	75	Dementia
SG17	F	51	Moldova	Single	Middle school	No	10	7	House with friends	Yes	M	67	Heart attack
TF18	2	51	Ukraine	Married	Middle school	No	7	9	House of patient	Yes	M	76	Alzheimer
UE19	2	49	Ukraine	Single	High school	No	9	7	House with friends	Yes	F	74	Visually impaired

Abbreviations: F, female; HCW, home care worker; M, male.

### 3.4 Data analysis

Every interview was transcribed verbatim, and the field notes made during the interview were integrated with the interview transcripts. Each interview transcript and the associated field notes were first read in their entirety to obtain an overview of the participant's experiences. Consistent with the method described by Cohen et al. (2000), each researcher then reread each transcript line by line and assigned broad themes to the various passages of the text. Indeed, Cohen et al. (2000) defined three phases in the data analysis. The first phase is called “immersing oneself in the data,” which aim is the establishment of an initial interpretation of data that will drive later coding of the data in subsequent phases of analysis. In this first phase, researchers identify the essential characteristics in the data from each interview. Consequently, researchers proceeded with second phase, called data transformation or data reduction. This process is like editing, where researchers deleted digressions that could be clearly off topic. In the third phase of the data analysis, data were subjected to the line‐by‐line coding, necessary for thematic analysis. Thereafter, the researchers compared their extracted themes. No discrepancies among the researchers' findings were found at this stage. The participants were informed of the identified themes in a second meeting (via video call) to confirm the extracted themes. They confirmed all the themes and did not add any further to the experience they had told. This process, called also member checking, results being fundamental in Cohen's phenomenology (Cohen et al., 2000). Indeed, as described by Cohen et al. (2000) and Lincoln & Guba (1985), themes should be verified with participants to ensure that the themes appropriately capture the meaning that participants sought to convey. Disagreements in interpretation should send the researcher back to the field text for clarifications.

### 3.5 Rigor

We also adopted Lincoln and Guba's criteria (Lincoln & Guba, 1985) for qualitative research to further guarantee the scientific rigor of this study. Continuing sampling with data collection until the same is saturated has ensured credibility. In qualitative research, credibility corresponds to internal validity and is a criterion for ensuring that the study examines what it is intended to (Lincoln & Guba, 1985; Shenton, 2004). In our study, we used a technique called triangulation. Triangulation method involves the participation of two or more researchers in the same analysis to provide multiple observations and conclusions (Lincoln & Guba, 1985). This method can bring both confirmation of findings and different perspectives, adding breadth to the phenomenon of interest. Triangulating the analysis among the researchers ensured the reliability criterion. Asking the participants for confirmation about themes extracted and the use of bracketing by researchers made it possible to satisfy the criterion of confirmability. Reporting a thorough description of the experience of our participants with a description of their sociodemographic characteristics ensured compliance with the transferability criterion. Transferability is communicated, in part, through description of sampling factors such as geographical location of the study, number and characteristics of participants, and the time frame of data collection and analysis (Shenton, 2004). Such descriptions also contribute to the credibility of the results and readers' determination of transfer to their and other contexts.

### 3.6 Ethical considerations

The institutional review board of University of Rome Tor Vergata approved the study. The study conforms to the principles outlined in the Declaration of Helsinki. Before signing the informed consent form, the HCWs were informed of the study's aim and nature and advised that their interviews would have been used with confidentiality, ensuring that no single participants could not be traced in the reporting study. Confidentiality was addressed during the research planning and at three points during the research process: data collection, data cleaning and dissemination of research. At the data collection, researchers make assurances of confidentiality, typically via consent form statements. During the data cleaning, researchers removed identifiers to create a clean data set, which did not contain information such as a name or address. In the dissemination of research, participants were coded. The participants were also told that they could withdraw from the study at any time. They did not receive any type of payment for contributing to the study.

## 4 RESULTS

From the analysis of the data, four principal themes emerged: (1) “I found myself alone”; (2) from invisibility to visibility; (3) a fear of getting sick and infecting others; and (4) “Health or work? That is the question.”

### 4.1 Theme 1: “I found myself alone”

All the study participants said that they remembered their feeling of sudden isolation during the first wave of the pandemic, particularly because of the government measures of total restriction to arrest the spread of COVID‐19. Their relationships with their compatriots and/or with the other members of their clients' families were suddenly cut. As they continued to work during the pandemic, the people who were normally around them before the pandemic disappeared. The members of their clients' families, who often used to help them accomplish household chores, suddenly stopped doing so. Suddenly, all responsibilities fell into their laps, making them feel alone and isolated in their new reality, quite different from what they had been used to.

At the beginning … I suddenly found myself alone [sitting, with the palms of her hands at chest height, parallel to the legs, she spreads her arms and hands outwards], with no one ever coming home. Suddenly, everything depended on me … it was just my client and I. I had to think of everything …. (AZ01)

My patient and I found ourselves alone. The family was not really completely gone, but I rarely saw them …. At that time, I worked even at night because I couldn't go home to my family as I also had to protect them from the virus in case I had been infected by it. That made me feel even more alone …. I had to think here and at work, to assist, but I had to think about them too …. I was sad and I felt bad. I felt alone. (MM11)

The network of friendships that had been established by the participants also suffered due to the pandemic and the restrictions that were imposed by the government to limit the spread of the virus. Therefore, what they thought they had gotten a foothold on proved to be unstable after all, which increased their sense of isolation.

For the first time [with her right hand half open, she makes a gesture that can be likened to throwing something behind], suddenly I was alone and had to look after myself and my client. I had to do everything myself, even more than before …. I felt really alone then because even in my own house, where I lived, no one left his or her room, and nobody wanted to talk too much because we all didn't know if everyone used masks where they worked …. Sometimes, you didn't know if you were alone in the house or not.(BV02)

Suddenly, even your friends didn't want to see you, you know … We decided to live together so we could share the expenses, and we often ate together in the evening or did some things together. When the pandemic began, it seemed that they too were far away …. We sometimes spoke by videophone, almost as if they were in Ukraine, like my real family …. Even with the other HCWs … we sometimes went shopping together or did chores together, you know, for company … but ever since the pandemic began, nobody had wanted to be with other people . (IO09)

### 4.2 Theme 2: From invisibility to visibility

Despite their sense of total isolation, the study participants found a new social consideration of their role. They felt appreciated and noticed a new or increased interest in them and their work. They understood that their clients were often more vulnerable than others, and they saw how their work was being viewed in a different light not only by the members of their clients' families but also by healthcare professionals. Suddenly, they perceived people's recognition of the strategic importance of their role, which people had seen in a different light up to that moment. The new attention to their work was well received. All the participants declared that they no longer felt “invisible” in the community because they noted that many people started to understand the importance of their role, especially during this pandemic.

From the almost invisible people we were before, we became more important. It's as if they had turned a light on us …. Now, in addition to everything that we did before, we also saw other symptoms, and we checked more. It seems that our clients' families and friends saw this.(OM 13)

I also see now how everyone is more interested in me, in what I'm doing. People compliment me regarding how I do my job, how I behave [in a sitting position, he suddenly straightens his back and widens his shoulders; a smile appears on his face]. Now, everyone is thanking me. For example, when the doctor called me to ask about my client's medicines, he also wanted to know how my clients and I were and how I organized my tasks …. (ES05)

After the first wave of COVID‐19, however, it seems that the role of HCWs has been slightly downsized.

Things now almost seem to be going back to how they were before … The work that I do is always the same. I stay close to my client, I help him, I work at home, I take care of him … It seems, however, that my ideas, my thoughts, and I are disappearing again. (FR06)

### 4.3 Theme 3: A fear of getting sick and infecting others

The study participants described their fear of acquiring COVID‐19 and of making people around them (e.g., their family and friends) sick. They described how their fear of being infected and of infecting others, especially their clients and/or their families, was always constantly present in their minds. They also felt that they could not always comply with the recommendations given by the government to protect themselves from being infected, and that this would not always be because they were negligent.

Before, I was much more afraid not only for myself but also for those who were close to me. Every day I took public transport to go to work …. It was therefore impossible to be careful …. I always wore a mask, but keeping a wide distance between two people was impossible. I thought that if my client got sick, especially at the beginning, the fault could only be mine even when I just went to buy medicines or other things for my client's house. I was always afraid of getting sick and making him sick.(CU03)

At first, I was afraid of bringing the disease not only to work but also to my home, to my son.…I was the one who went to work, did the shopping and went to get the medicines and everything else. I was responsible if something happened because I was the only one who was going out …. I was therefore always worried that if my client or my son got sick it would be my fault [puts her hand on her chest].(NN12)

### 4.4 Theme 4: “Health or work? That is the question”

The study participants described how many of the choices they had made during the first wave of the present pandemic were a compromise between safeguarding their and others' health and the need to work. It was as if there was a tug of war between their fear of contracting the virus and of conveying it to their clients and loved ones on the one hand and knowing they needed to work on the other. However, as HCWs often do not have a regular contract with their clients with the specific protections provided by law, the economic aspect prevails over their personal feelings.

I was really afraid, and I still am, but we have to work; otherwise, we can't stay alive [she puts her right hand on her belly and then moves it quickly up and down]…. I came here to give my family a better future, and for this I allowed myself to face many difficult things, and I'm still doing that now …. I've turned my back on my personal protection, and I go on ….(LN10)

The fear was so much, very much, and now it's still there … But we have to work; otherwise, we can't stay alive [she puts her right hand on her belly and then moves it up and down in quick succession] … I came here to give my family a better future, and for this I allowed myself to face many difficult things, and I'm still doing that now …. I've turned my back on my personal protection, and I go on. (LN10)

Fear was always there, but what should I do? Health or work? That is the question. If you reject the work, then how do you stay alive?(DT04)

## 5 DISCUSSION

In this study, we explored the lived experiences of HCWs in Italy who cared for patients during the first wave of the COVID‐19 pandemic. To the best of our knowledge, this study is only the second to focus on the HCWs' lived experiences during the present COVID‐19 pandemic (Sterling et al., 2020) and the first to be conducted in a European country. We discovered important issues that should be considered by investigators, clinicians, administrators, and the public. Despite the present and future importance of HCWs, little is known about their lived and work experiences, especially the crucial role that they have played so far in this pandemic.

The first theme that we identified was “I found myself alone.” The study participants described the feeling of isolation that they had experienced during the first wave of the pandemic because of the restrictive measures taken by the government to arrest the spread of the virus. In many cases, the participants found themselves isolated in their client's home, without any support. In Italy, it is very common for HCWs to live 24 h a day with their clients (Bilotta & Vergani, 2008). The measures issued by the Italian government to contain the spread of the COVID‐19 pandemic led to a drastic reduction in visits to friends and family. As described by some of the HCWs interviewed in this study, it seemed as if everything depended on them. This feeling of being abandoned by everyone, not having support, and having the overwhelming responsibility (“*everything weighs on me*”) of having to take care of their client without ever letting go may have increased the burden and loneliness of HCWs (Del‐Pino‐Casado et al., 2018; Dich et al., 2019; Simard & Volicer, 2020). The feelings of being overburdened and loneliness can have many deleterious consequences; it has been shown that higher burden levels can decrease HCWs' quality of life (Lo et al., 2019; Perpina‐Galvan et al., 2019).

The feeling of no longer being invisible was the second theme that was identified in the study participants' interviews. HCWs constitute an invisible and vulnerable group in society, especially when they are migrant workers. In many countries, they are considered “guest workers” and will not be given the benefit of citizenship at any time in the future. HCWs are usually “invisible” in society (Ahonen et al., 2010), and many family employers are also unaware that domestic labor and caregiving may have negative impacts on one's health. Intimacy with one's employers and a variety of psychosocial, physical, biological, and chemical conditions have been shown to be important risk factors for the health and safety of HCWs (Muramatsu et al., 2019; Orrenius & Zavodny, 2009). A live‐in regimen could mean more working hours, lack of privacy, social isolation, and little rest (Hewko et al., 2015).

However, for many, this pandemic represented an opportunity for change (“no longer invisible,”) as the study participants found a new social consideration of their role. They felt appreciated and perceived a new or increased interest in them and their work from the members of their clients' families and from health professionals. Contrary to what was found in this study, however, Sterling et al. (2020) observed that the HCWs in their study still felt invisible to the healthcare community and society during the present pandemic despite their efforts to keep their clients healthy and safe.

The fear of getting sick or infecting others, especially their clients and their families, was a constant feeling among our sample of HCWs. Fear is an adaptive response in the presence of danger. With the spread of COVID‐19, national polls have indicated a sharp increase in fear and worries relating to the virus (Arora et al., 2020). In previous studies on healthcare professionals (Apisarnthanarak et al., 2020; Taylor et al., 2020), fear was found in almost all the participants. Researchers (Taylor et al., 2020) have observed that people's fear of acquiring COVID‐19 could be related to different topics, including fear of danger and contamination, fear of the social and economic consequences of such, coronavirus‐related xenophobia, compulsive checking and reassurance seeking, and traumatic stress symptoms. However, although the feeling of fear can be common during a pandemic or dangerous event, if it is not kept in check, it may have serious health consequences. For example, previous studies have identified the consequences of fear at the individual level, such as mental health problems, anxiety, and phobias (Shin & Liberzon, 2010), or at the societal level, such as panic shopping or xenophobia (Sim et al., 2020). Once again, it is evident how strong the impact of this pandemic has been on the study participants, making them increasingly vulnerable. These findings suggest the need to provide psychological support not only for healthcare professionals but also for HCWs.

Not knowing whether to give more importance to their health or to their work was the final theme as identified in the interviews with the study participants. One participant described this theme in a somewhat Shakespearean form: “Health or work? That is the question,” which represents a real problem. Having to decide whether to prioritize health or work puts one at an even greater risk. However, it is not news that HCWs typically have precarious contracts (Hill et al., 2019), as in Italy, HCWs are often underpaid and living in difficult conditions (Bilotta & Vergani, 2008). For migrants, such work often represents the only form of sustenance available. Our findings in this study showed that despite the risks involved for fear of losing their jobs and not being able to support their families, many HCWs worked and thus set aside their health aside for work. In addition, the living conditions and multigenerational households of migrant HCWs may increase their risk of being infected with COVID‐19.As described in the literature (Burstrom & Tao, 2020), work‐related exposure is heightened for people in occupations that cannot work from home and that entail physical proximity to or direct contact with other people. Precarious employment and a lack of social insurance are also more common among low‐income earners, which can limit their financial ability to stay home during sickness.

This study has several implications. First, the results obtained from the interviews highlighted the extent to which HCWs are at risk from a psychological viewpoint and from the viewpoint of acquiring the disease. Second, it is known that HCWs have an effect on their clients' perceived quality of life; thus, anything that affects HCWs may also have implications for their clients. The COVID‐19 pandemic has underscored the loneliness of HCWs. Loneliness can increase the risk of depression, alcoholism, suicidal thoughts, aggressive behaviors, anxiety, and impulsivity (Cacioppo et al., 2015; Yanguas et al., 2018). Some studies found that loneliness is also a risk factor for cognitive decline, recurrent stroke, obesity, elevated blood pressure and mortality (Hawkley et al., 2010; Lara et al., 2019). Future studies should therefore longitudinally analyze the impact of the current COVID‐19 pandemic on HCWs' physical and mental health.

The HCWs' mental health influences their performance and therefore the health of the assisted subjects and has repercussions on the family unit. Nurses and physicians also have a responsibility toward the preparation of HCWs. Understanding how they dealt with the COVID‐19 pandemic will allow nurses to understand how to relate to the HCWs, increasingly of reference for older patients and their families.

## 6 CONCLUSION

This study provided a comprehensive and in‐depth understanding of the lived experiences of HCWs caring for patients during the current COVID‐19 pandemic. As observed in our study, the Italian HCWs' psychological health has been severely affected by the first wave of the COVID‐19 pandemic. Countries should pay greater attention to HCWs and should provide them with greater work security. These results should induce researchers and clinicians to give greater visibility to HCWs by undertaking longitudinal research projects aimed at analyzing their quality of life and the effects of their work on them. Greater attention should be paid to HCWs and to their role toward patients and the family unit.

## 7 RESEARCH LIMITATIONS

This study had several limitations. First, it focused on only one European country (Italy), which limits its generalizability to other countries that may have different labor policies for HCWs. Another limitation is possible selection bias. Many subjects, especially migrant workers with precarious jobs and who fear work repercussions, could have chosen not to participate in this study, and only those who had more favorable working conditions could have chosen to participate. Finally, this study used video call interviews, which, as described in the literature, could substitute for the classic face‐to‐face interviews. In reality, however, the video call interviews in this study might have failed to capture nonverbal information, which could have been easier to capture in face‐to‐face interviews. Many of the authors are experts in qualitative interviewing and analysis and have instructed less experienced colleagues. Two pilot interviews were conducted to ensure correct familiarity with data collection and data analysis.

## CONFLICT OF INTEREST

The authors declare that they have no conflicts of interests.

## AUTHOR CONTRIBUTIONS

*Study design*:: Silvio Simeone, Ercole Vellone, Madeline R. Sterling. *Data collection*: Silvio Simeone, Michele Virgolesi, Gianluca Pucciarelli. *Data analysis*: Silvio Simeone, Ercole Vellone, Michele Virgolesi, Madeline R. Sterling. Rosaria Alvaro, Gianluca Pucciarelli. *Manuscript writing*: Simeone, Ercole Vellone, Michele Virgolesi, Madeline R. Sterling. Rosaria Alvaro, Gianluca Pucciarelli.

## Data Availability

The data that support the findings of this study are available on request from the corresponding author. The data are not publicly available due to privacy or ethical restrictions.
